# The influence of ESR1 polymorphisms on selected hormonal, metabolic and mineral balance markers in women with hyperandrogenism

**DOI:** 10.1038/s41598-022-17383-w

**Published:** 2022-11-16

**Authors:** Izabela Uzar, Anna Bogacz, Elżbieta Sowińska-Przepiera, Katarzyna Kotrych, Marlena Wolek, Tadeusz Sulikowski, Adam Kamiński

**Affiliations:** 1 Department of Pharmacology and Pharmacoeconomics, Pomeranian Medical University in Szczecin, 71-230 Szczecin, Poland; 2 Department of Stem Cells and Regenerative Medicine, Institute of Natural Fibers and Medicinal Plants, 62-064 Plewiska, Poland; 3 Department of Endocrinology, Metabolic Diseases, and Internal Diseases, Pomeranian Medical University in Szczecin, 71-252 Szczecin, Poland; 4 Department of General and Dental Radiology, Pomeranian Medical University in Szczecin, 70-111 Szczecin, Poland; 5 General, Mini-Invasive and Gastroenterogical Surgery Clinic, Pomeranian Medical University in Szczecin, 71-252 Szczecin, Poland; 6 Department of Orthopedics and Traumatology, Independent Public Clinical Hospital No. 1, Pomeranian Medical University, 71-252 Szczecin, Poland

**Keywords:** Genetics, Molecular biology, Endocrinology

## Abstract

Hyperandrogenism is the most common endocrine disorder in women, characterized by an imbalance in normal estrogen and androgen levels in the blood. Androgens influence bone mineral density, body mass composition, muscle mass, mental state, and the regulation of sexual function.. The aim of the study was to assess the effect of estrogen receptor α gene (ESR1) polymorphisms on selected markers of bone metabolism and hormonal parameters in women with hyperandrogenism. The study group included 80 young women with hyperandrogenism who underwent measurements of bone mineral density (BMD), and determination of hormonal and metabolic parameters. Enzyme immunoassays were used to measure leptin, sRANKL (soluble receptor activator of nuclear factor-kB ligand), osteoprotegerin and 25-OH vitamin D total levels. An analysis of ESR1 gene polymorphisms was performed using the real-time PCR method. A relationship was demonstrated between the concentration of free estradiol (FEI) and the concentration of 17-OH-progesterone, and the ESR1 gene polymorphisms: rs3020314 (p = 0.031, p = 0.026 respectively) and rs1884051 (p = 0.033, p = 0.026 respectively). In conclusion, the ESR gene polymorphisms may be associated with hormonal disturbances in the concentration of estrogens and androgens, in hyperandrogenism in young women which may indirectly affect bone mineral density. However, no statistically significant relationships between the studied polymorphisms and the selected parameters of mineral metabolism have been demonstrated..

## Introduction

Hyperandrogenism is the most common endocrine disorder in women and affects 5–10% of women of reproductive age^1^. It is characterized by a disturbance in the correct balance between the level of estrogens and androgens in the blood. Androgens play an important role in the hormonal balance of women. They affect bone mineral density, body mass composition, muscle mass, mental state, and the regulation of sexual functions. However, an excess in the female body leads to a wide range of hormonal and metabolic disorders. In women, androgens are produced in the ovaries and adrenal glands: testosterone (T), dihydrotestosterone (DHT), androstenedione (A), and dehydroepiandrosterone sulfate (DHEAS). Only two of them, testosterone and dihydrotestosterone, bind to the androgen receptor and show a direct androgenic effect. The remaining androgens are converted peripherally to testosterone, which provides about 50% of this hormone to the body. The remaining 50% comes from ovarian and adrenal production. DHT is made from testosterone and has the strongest androgenic effects^2,3^.

Hyperandrogenism is a disorder related to the excessive production of androgens, manifested by the appearance of masculine features and metabolic changes as well as a decrease in feminine features. The characteristic symptoms are male excess hair, skin changes (seborrhea, acne), male pattern baldness, an increase in muscle mass, enlargement of the clitoris, deepening of the voice, depressive disorders, libido disorders, menstrual disorders, atrophy of the mammary glands, metabolic disorders (hyperinsulinemia, obesity, changes in lipid profile)^4,5^. The etiopathogenesis of hyperandrogenism is complex and may be multifactorial. The most important marker of hyperandrogenism in young women are menstrual disorders, hirsutism, acne and androgenic alopecia. Hyperandrogenism affects 5–10% of women of childbearing age. Idiopathic hyperandrogenism and idiopathic hirsutism account for 5–20% of cases in which peripheral conversion to androgens takes place, similar to obesity and with an increase in 5α-reductase activity. Determining the source of androgen excess is a diagnostic and therapeutic challenge, but early diagnosis and treatment of androgenization can avoid long-term health effects.

One of the causes may often be functional ovarian hyperandrogenism (FOH) preceding the onset of full-blown polycystic ovary syndrome (PCOS). This is a disorder of ovarian steroidogenesis leading to hyperandrogenemia^6,7^. Estrogens play a key role in the functioning of the reproductive organs of a woman and have a systemic function, regulating the metabolism of lipids, carbohydrates, bone mineralization and the activity of the circulatory system^8^. As steroid hormones, estrogens have the ability to penetrate cell membranes and bind to the intracellular estrogen receptor (ER). There are two main types of estrogen receptors: ERα and ERβ. Estrogens complexed with the receptor bind to a specific region of DNA, changing the expression of genes, and not the activity of a single gene^9^. One of the main functions of estrogens is to participate in the bone mineralization process. The effect on bone growth and mineralization depends on the type of receptor through which estrogens act. Osteoblasts are activated by acting on the ER-α receptor. On the other hand, the effect of estradiol on osteoclasts is indirect, through regulatory factors^10–12^. The effect of estrogens on the bone mineralization process is also indirect, by stimulating the secretion of growth hormone (GH) by the pituitary gland, which increases the level of insulin-dependent growth factor (IGF-1)^13^. Increasing the IGF-1 level increases the level of bioavailable estradiol (FEI) by lowering the amount of sex hormone binding globulin (SHBG) and reduces the effects of GH and IGF-1 on bone tissue^14^. The ER-α receptor also seems to be involved in the transmission of mechanical stimuli to bone tissue^15^. Bioavailable estradiol is a fraction unbound with the SHBG protein, biologically active. Androgens constitute an additional pool for the production of estrogens as a result of aromatization under the action of aromatase—an enzyme included in the cytochrome P450, encoded by the CYP19 gene, present in peripheral, extra-glandular tissues (adipose tissue, muscles, liver)^16^.

The effectiveness of EP therapy is mostly determined by the ability to respond to hormonal substitution. This was confirmed both in studies^17–19^ and in experiments by other authors^20–22^. All suggesting the involvement of the ER-α gene polymorphisms in determining response to oestroprogestagen therapy.

The existing literature has not found a study that would assess the influence of genetic factors on changes in bone mineral density following hormonal treatment. A review of the literature data on the genetic determinants of mineral density in women indicates significant discrepancies in the results of the studies conducted so far. In one experiment, pre-pubertal girls with the PvuII ERα gene PvuII genotype had an almost five-fold higher relative risk of spine fractures and bone mass deficiencies compared to their peers with the pp genotype^23^. However, studies of postmenopausal women receiving hormone therapy have not determined which of the ERα genetic variants is a predictor of a worse response to treatment in the context of normalization of bone mineral density. Depending on the study, an unfavorable predictor was having the haplotype xxPP^24,25^, genotype XX^20,26^ or genotype PP^20^. Other authors found no significant relationship between the ER α genotype and the response to hormone therapy^21,22,27^.

It is now known that hyperandrogenism is a multi-gene disorder influenced by epigenetic and environmental factors. The search for genetic determinants aims to improve diagnostics and therapeutic possibilities. Considering the role of estrogens in maintaining the balance of sex hormones in the female body, it can be assumed that genetic polymorphisms related to estrogen receptors may play an important role in correct hormonal regulation and the type of disorders.

We selected three SNPs (rs3020314, rs2077647 and rs1884051) on the basis of the following criteria: minor allele frequency > 0.2, fuctional relevance and importance, SNPs significantly associated with BMD in previous studies.

The ESR1 gene polymorphisms are often analyzed in the literature for their influence on bone mineral density. It is known that numerous polymorphisms of the ESR1 gene are associated with an increased risk of osteoporosis in postmenopausal women. Few reports concern bone mineralization disorders in young women with hyperandrogenism. A question arises whether, by influencing the hormonal balance of estrogens, they may also be of importance for bone mineral density in hyperanrogenism. The research undertaken is aimed at broadening the knowledge about the genetic determinants of mineral and metabolic disorders in young women with hyperandrogenism. Selected SNPs have already been studied earlier in terms of the risk of ostoporosis in postmenopausal women and in HIV-infected women. The aim of the study was to assess the effect of ESR1 gene polymorphisms on the increased risk of osteoporosis in women with hyperandrogenism.

## Methods

### Patients

The study was conducted from 2013 to 2015, consisted of 80 young women aged 18 to 35 with hyperandrogenism, attending the Department of Endocrinology, Metabolic Diseases, and Internal Diseases, at the Pomeranian Medical University in Szczecin (Poland). The analysis included the patients with idiopathic hyperandrogenism defined as the presence of clinical and biochemical hyperandrogenism in the absence of ovulatory dysfunction and sonographic features of polycystic ovaries, provided that secondary etiologies had been excluded^28,29^, who satisfied the following inclusion criteria: (1) caucasian race, (2) at least six months of amenorrhea preceded by at least three years of oligomenorrhea, (3) clinical symptoms of androgenization: acne, seborrhea, hirsutism (4) no actual history of chronic pharmacotherapy, and (5) a lack of major abnormalities on physical examination. The exclusion criteria were: (1) polycystic ovary syndrome, congenital adrenal hyperplasia or premature ovarian failure diagnosed on the basis of medical history, gynecological exam and laboratory tests, (2) low birth weight or preterm birth, (3) at least one confirmed episode of an eating disorder, (4) poor diet during childhood or puberty, (5) episodes of impaired growth and body mass gain, (6) extensive participation in sports that may have influenced bone mineralization, (7) metabolic disorders that may be associated with decreased bone mineralization, (8) prolonged use of stimulants or drugs that may affect bone metabolism, and (9) familial history of osteoporosis.

Hirsutism was assessed according to the Ferriman-Gallwey scale (≥ 8 points). All patients also had elevated androstenedione which is a precursor of androgens, leptin and BMI above normal.

The lack of control group is a potential limitation of the study. Initially, women were recruited into the study as a test and control group. However, careful evaluation of the patients and long-term analysis of their health showed the absence of chronic diseases and the absence of organic causes of the reported disorders identified in the course of diagnostics. Menstrual disorders were considered to be transient and therefore both study groups were combined.

The study was approved by the Ethical Committee of the Pomeranian Medical University in Szczecin (no. KB-0012/115/15 of 16 November 2015). The study was conducted in accordance with the Helsinki Declaration (1975, revised 2000). Written informed consent was obtained from all participants.

### Determination of serum concentrations for selected parameters

Blood samples were obtained in the fasted state at 8 am. Enzyme immunoassays (ELISA—DRG International, Inc., USA ) were used to measure leptin—intra- and inter-assay CV of 4,6% and 6,2% respectively, sRANKL (soluble receptor activator of nuclear factor-kB ligand)—intra- and inter-assay CV 0,9 and 9,3% respectively, osteoprotegerin—intra- and inter-assay CV 3,0 and 5,0% respectively and 25-OH vitamin D total—intra- and inter-assay CV 3,7 and 7,13% respectively levels. Serum concentrations of parathyroid hormone and calcitonin were determined using the chemiluminescent method (Immulite 1000, Siemens). Electrochemiluminescence immunoassays (Cobas, Roche Diagnostic, USA) were used to measure follicle stimulating hormone (FSH), luteinizing hormone (LH), sex hormone-binding globulin (SHBG), total testosterone (TT), androstenedione, 17-hydroxyprogesterone, estradiol, prolactin (PRL), dehydroepiandrosterone sulfate (DHEA-SO4), glucose and insulin. The free androgen index (FAI = TT/SHBG × 100%) was used to evaluate free testosterone concentrations.

Analytical sensitivity of the tests: parathyroid hormone 3.1 pg/ml, calcitonin 2.0 pg/ml, 25-OH vitamin D total 5.6 nmol/l, osteoprotegerin 0.14 pmol/l, leptin 2.0 ng/ml, sRANKL 0.5 pmol/l. Elisa tests assays were performed as per the manufacturer’s instructions and were quality controlled using the manufacturer’s two-level control set. Samples were run in duplicate. The performance of the microplate reader and microplate washer used for ELISA determinations and precision was controlled with Pathozyme Elisa Sure kit (Omega Diagnostics, UK).

### Bone mineral density (BMD) measurements

Bone mineral density (BMD) measurements were taken at the Endocrinology Clinic at Clinical Hospital No.1, Pomeranian Medical University in Szczecin. BMD was measured in the lumbar spine from L2 to L4 vertebrae with the use of DEXA (Dual Energy X-ray Absorptiometry). Densitometry was performed using the LUNAR DPX 100 camera (Lunar Corp., Madison, USA). All patients were imaged using the same DEXA Lunar in order to minimize intermachine variabilit*y.* Quality assurance on this device was performed as recommended by the International Society for Clinical Densitometry (ISCD)^30^.

### ESR1 genotyping

Blood samples were collected at the Department of Endocrinology, Metabolic Diseases, and Internal Diseases, Pomeranian Medical University. ESR1 genotyping was performed in the Clinical Laboratory at the Department of Endocrinology, Metabolic Diseases, and Internal Diseases, Pomeranian Medical University. Genomic DNA was extracted from peripheral blood using a QIAamp Blood Mini Kit (Qiagen GmbH, Hilden, Germany) according to the manufacturer’s protocol. ESR1 gene polymorphisms rs3020314, rs2077647 and rs1884051 were selected as per the NCBI SNP database http://www.ncbi.nlm.nih.gov/SNP and determined by Real-Time PCR using LightCycler 480 and TaqMan^®^ SNP Genotyping Assays (Thermo Fisher Scientific, Waltham, USA, Assay ID: C_11555860_10, C_11414978r_10, C_11918415_10 respectively). Real-time PCR was performed in a 20 μl reaction mixture according to the manufacturer’s protocol: initial denaturation at 95ºC for 10 min and 40 cycles as follows: denaturation at 95 °C for 15 s, annealing at 60ºC for 1 min and cooling at 40 °C for 30 s.

### Statistical analysis

All statistical analyses for this study were performed using SPSS Statistics 17.0 for Windows. The chi-square test was used to calculate that the genotype prevalence and allele frequencies were in the Hardy–Weinberg equilibrium. We evaluated the effect of the ESR1 polymorphism on selected biochemical and clinical parameters. Correlation analysis between the genotypes and the clinical parameters was conducted using one-way ANOVA. A *p*-value less than 0.05 was considered to indicate statistical significance.

All methods were carried out in accordance with relevant guidelines and regulations.

## Results

Comparing the frequency distribution of individual genotypes for the analyzed polymorphisms in women with hyperandrogenization, a higher frequency of TT genotype (51.25%), and the lowest CC genotype (11.25%) for the ESR1 rs3020314 polymorphism were observed. In the case of the rs2077647 polymorphism of the ESR1 gene, a higher frequency of the CT genotype (48.75%) was observed compared to the CC (25%) and TT (26.25%) genotypes. In patients with ESR1 rs1884051 polymorphism, the wild type AA genotype was dominant (53.75%), and the mutant GG genotype (10%) was the lowest (Table 1).

**Table 1:** The frequency of alleles and genotypes of the ESR1 polymorphisms in women with hyperandrogenism.

ESR1 rs3020314			ESR1 rs2077647			ESR1 rs1884051		
Genotype	Observed value n (%)	Epected value (%)	Genotype	Observed value n (%)	Expected value (%)	Genotype	Observed value n (%)	Epected value (%)
CC	9 (11.25)	9	CC	20 (25.00)	24.38	AA	43 (53.75)	51.67
CT	30 (37.50)	42	CT	39 (48.75)	50.00	AG	29 (36.25)	40.42
TT	41 (51.52)	49	TT	21 (26.25)	25.62	GG	8 (10.00)	7.91
Total	80 (100)	100	Total	80 (100)	100	Total	80 (100)	100
Allele			Allele			Allele		
C	48 (30)	–	C	79 (49.38)	–	A	115 (71.88)	–
T	112 (70)	–	T	81 (50.62)	–	G	45 (28.12)	–
Total	160 (100)	–	Total	160 (100)	–	Total	160 (100)	–

In this study, the frequency of individual genotypes for the polymorphisms of the ESR1 rs1884051, rs2077647 and rs30220314 gene was determined and compared with clinical parameters. In the case of the ESR1 rs3020314 polymorphism, a significantly higher level of free estradiol (p < 0.05) was demonstrated in CC homozygotes (13.14 ± 4.29 pmol/nmol) compared to CT heterozygotes (5.72 ± 1.12 pmol/nmol) and TT homozygotes (6.9 ± 0.86 pmol/nmol). Also, the concentration of 17-OH-progesterone was significantly higher (p < 0.05) in CC homozygotes (2.45 ± 1.05 ng/ml) compared to the CT (1.36 ± 0.14 ng/ml) and TT (1.14 ± 0.12 ng/ml) genotypes (Table 2).

**Table 2:** Analysis of female hormonal parameters in relation to ESR1 gene polymorphisms in patients with hyperandrogenism.

Parameter	ESR1 rs3020314	Mean ± SEM	*P*	ESR1 rs2077647	Mean ± SEM	*P*	ESR1 rs1884051	Mean ± SEM	*P*
Estradiol (pg/ml)	CC	89.88 ± 27.14	0.053	CC	67.05 ± 12.78	0.177	AA	52.88 ± 5.17	0.055
	CT	45.41 ± 7.47		CT	49.48 ± 5.23		AG	45.34 ± 8.20	
	TT	53.24 ± 5.46		TT	45.69 ± 6.55		GG	89.88 ± 27.14	
FEI (pmol/nmol)	CC	13.14 ± 4.29	**0.031**	CC	9.21 ± 1.83	0.095	AA	6.82 ± 0.83	**0.033**
	CT	5.72 ± 1.12		CT	6.96 ± 1.07		AG	5.75 ± 1.20	
	TT	6.9 ± 0.86		TT	4.87 ± 0.84		GG	13.14 ± 4.29	
Prolactin (ng/ml)	CC	17.41 ± 5.62	0.972	CC	14.37 ± 1.84	0.120	AA	17.64 ± 2.54	0.997
	CT	17.05 ± 1.71		CT	16.30 ± 1.20		AG	17.38 ± 1.85	
	TT	17.89 ± 2.69		TT	22.74 ± 5.07		GG	17.40 ± 5.62	
17-OH-Progesterone (ng/ml)	CC	2.45 ± 1.05	**0.026**	CC	1.67 ± 0.55	0.551	AA	1.15 ± 0.11	**0.026**
	CT	1.36 ± 0.14		CT	1.24 ± 0.13		AG	1.36 ± 0.15	
	TT	1.14 ± 0.12		TT	1.32 ± 0.18		GG	2.45 ± 1.05	
LH (mIU/ml)	CC	10.52 ± 1.03	0.709	CC	7.27 ± 1.25	0.109	AA	10.82 ± 1.52	0.792
	CT	9.03 ± 1.25		CT	10.66 ± 1.55		AG	9.16 ± 1.42	
	TT	10.98 ± 1.61		TT	13.09 ± 2.54		GG	10.52 ± 1.03	
FSH (mIU/ml)	CC	4.83 ± 0.31	0.267	CC	7.9 ± 2.77	0.856	AA	10.80 ± 2.81	0.295
	CT	5.61 ± 0.33		CT	8.08 ± 2.28		AG	5.52 ± 0.35	
	TT	11.04 ± 2.96		TT	10.13 ± 4.15		GG	4.83 ± 0.31	

*FEI* Free Estradiol Index, *LH* luteinizing hormone, *FSH* follicle stimulating hormone.

Significant values are given in bold.

The presence of ESR1 rs2077647 polymorphism was associated with a significantly higher concentration of DHEA-SO4 (p < 0.05) in CT heterozygotes (314.72 ± 24.92 µg/dl) compared to TT homozygotes (233.54 ± 32.64 µg/dl) and CC homozygotes (203.02 ± 28.43 µg/dl). The rs2077647 polymorphism was also associated with a statistically significantly higher concentration of vitamin 25-(OH)D (p < 0.05) in TT homozygotes (26.23 ± 1.68 ng/ml) compared to the CT (20.90 ± 1.13 ng/ml) and CC (18.62 ± 1.43 ng/ml) genotypes (Tables 3 and 4). The levels of other parameters: estradiol, prolactin, 17-OH-progesterone, LH, FSH, SHBG, testosterone, BAT%, FAI%, androstendione, vitamin D, calcitonin, parathyroid hormone, osteoprotegerin, sRANKLt, leptin, BMD total, BMD L1-L4, T-score, Z-score, BMI, BMC, AG, TBS and glucose in women with hyperandrogenism in relation to the distribution of genotypes of the ESR1 rs3020314 polymorphism did not show any significant statistical differences.

**Table 3:** Analysis of hormonal parameters in relation to ESR1 gene polymorphisms in patients with hyperandrogenism.

Parameter	ESR1 rs3020314	Mean ± SEM	*P*	ESR1 rs2077647	Mean ± SEM	*P*	ESR1 rs1884051	Mean ± SEM	*P*
Testosterone (ng/ml)	CC	0.53 ± 0.05	0.821	CC	0.39 ± 0.04	0.114	AA	0.49 ± 0.04	0.815
	CT	0.50 ± 0.05		CT	0.54 ± 0.04		AG	0.47 ± 0.05	
	TT	0.47 ± 0.04		TT	0.50 ± 0.06		GG	0.53 ± 0.05	
BAI %	CC	42.93 ± 6.01	0.973	CC	46.00 ± 3.55	0.198	AA	42.45 ± 2.50	0.987
	CT	41.83 ± 2.70		CT	43.25 ± 2.44		AG	42.01 ± 2.72	
	TT	42.61 ± 2.54		TT	37.62 ± 3.40		GG	42.93 ± 6.01	
FAI %	CC	1.83 ± 0.26	0.974	CC	1.96 ± 0.15	0.199	AA	1.81 ± 0.11	0.987
	CT	1.78 ± 0.12		CT	1.84 ± 0.10		AG	1.79 ± 0.12	
	TT	1.82 ± 0.11		TT	1.60 ± 0.15		GG	1.83 ± 0.26	
Androstendione (ng/ml)	CC	5.43 ± 0.89	0.087	CC	3.55 ± 0.48	0.420	AA	3.90 ± 0.31	0.096
	CT	3.10 ± 0.29		CT	4.23 ± 0.33		AG	3.86 ± 0.30	
	TT	3.79 ± 0.31		TT	4.19 ± 0.36		GG	5.43 ± 0.89	
DHEA-SO4 (μg/dl)	CC	332.80 ± 69.44	0.280	CC	203.02 ± 28.43	**0.018**	AA	244.76 ± 23.08	0.293
	CT	274.65 ± 25.94		CT	314.72 ± 24.92		AG	274.00 ± 27.13	
	TT	243.46 ± 23.73		TT	233.54 ± 32.64		GG	332.80 ± 69.44	
SHBG (nmol/l)	CC	54.15 ± 27.51	0.642	CC	33.95 ± 5.20	0.276	AA	40.57 ± 4.20	0.663
	CT	42.99 ± 7.26		CT	40.91 ± 6.92		AG	42.30 ± 7.64	
	TT	39.97 ± 4.21		TT	53.30 ± 9.79		GG	54.15 ± 27.51	

*BAI%* Body Adiposity Index, *FAI%* Free Androgen Index, *DHEA-SO4* dehydroepiandrosterone sulfate, *SHGB* sex hormone binding globulin.

Significant values are given in bold.

**Table 4:** Analysis of bone metabolism parameters in relation to ESR1 gene polymorphisms in patients with hyperandrogenism.

Paramater	ESR1 rs30220314	Mean ± SEM	*P*	ESR1 rs2077647	Mean ± SEM	*P*	ESR1 rs1884051	Mean ± SEM	*P*
Vitamin D (ng/ml)	CC	8.67 ± 4.33	0.591	CC	14.4 ± 3.11	0.266	AA	15.90 ± 1.79	0.602
	CT	15.47 ± 3.60		CT	13.79 ± 2.01		AG	16.14 ± 3.92	
	TT	16.20 ± 1.84		TT	19.89 ± 3.73		GG	8.67 ± 4.33	
25(OH)D (ng/ml)	CC	25.17 ± 3.60	0.241	CC	18.62 ± 1.43	**0.002**	AA	20.63 ± 1.08	0.279
	CT	22.21 ± 1.23		CT	20.90 ± 1.13		AG	22.56 ± 1.27	
	TT	20.64 ± 1.13		TT	26.23 ± 1.68		GG	24.78 ± 4.06	
Calcitonin (pg/ml)	CC	1.57 ± 0.44	0.797	CC	4.22 ± 2.91	0.267	AA	2.61 ± 1.32	0.841
	CT	1.77 ± 0.27		CT	1.78 ± 0.22		AG	1.80 ± 0.28	
	TT	2.69 ± 1.39		TT	1.19 ± 0.19		GG	1.65 ± 0.50	
PTH (pg/ml)	CC	39.86 ± 4.01	0.289	CC	41.03 ± 5.93	0.635	AA	43.70 ± 4.04	0.392
	CT	35.75 ± 3.18		CT	42.30 ± 3.61		AG	36.48 ± 3.21	
	TT	44.22 ± 4.17		TT	36.48 ± 3.70		GG	38.35 ± 4.21	
OPG (pmol/l)	CC	3.48 ± 0.37	0.638	CC	3.32 ± 0.15	0.477	AA	3.69 ± 0.21	0.879
	CT	3.48 ± 0.23		CT	3.71 ± 0.25		AG	3.53 ± 0.24	
	TT	3.76 ± 0.22		TT	3.75 ± 0.30		GG	3.60 ± 0.40	
sRANKL (pmol/l)	CC	255.48 ± 97.98	0.394	CC	227.68 ± 46.10	0.487	AA	189.73 ± 15.47	0.389
	CT	240.61 ± 41.41		CT	190.27 ± 18.60		AG	240.08 ± 42.96	
	TT	188.10 ± 16.17		TT	248.46 ± 54.89		GG	263.90 ± 110.68	
Leptin (ng/ml)	CC	16.84 ± 4.24	0.379	CC	24.86 ± 4.32	0.420	AA	22.35 ± 2.43	0.555
	CT	24.89 ± 2.68		CT	23.43 ± 2.20		AG	24.33 ± 2.81	
	TT	22.19 ± 2.54		TT	18.91 ± 3.07		GG	17.69 ± 4.71	

*25(OH)D* 25-OH vitamin D, *PTH* Parathormon, *OPG* osteoprotegerin, *sRANKL* soluble receptor activator of nuclear factor-kB ligand.

Significant values are given in bold.

Analysis of the ESR1 rs188405 polymorphism showed a significantly higher level of free estradiol (p < 0.05) in GG homozygotes (13.14 ± 4.29 pmol/nmol) compared to the AA (6.82 ± 0.83 pmol/nmol) and AG (5.75 ± 1.20 pmol/nmol) genotypes. The concentration of 17-OH-progesterone was also significantly higher in GG homozygotes (2.45 ± 1.05 ng/ml) compared to the AG (1.36 ± 0.15 ng/ml) and AA (1.15 ± 0.11 ng/ml) genotypes. Android to gynoid fat distribution coefficient was also significantly higher (p < 0.05) in GG (1.16 ± 0.03) homozygotes compared to AA (1.12 ± 0.04) and AG (0.95 ± 0.06) genotypes (Tables 2 and 5). The concentration of other parameters: prolactin, LH, FSH, SHBG, testosterone, DHEA-SO4, glucose, insulin, vitamin D, vitamin 25(OH)D, calcitonin, parathyroid hormone, osteoprotegerin, sRANKL, leptin and other parameters: BAT%, FAI%, BMD total, BMD L1-L4, T-score, Z-score, BMI, BMC, VF and TBS in women with hyperandrogenism in relation to the distribution of genotypes of the ESR1 rs1884051 polymorphism did not show any significant statistical differences (Tables 2, 3, 4, 5, 6).

**Table 5:** Analysis of clinical parameters in relation to ESR1 gene polymorphisms in patients with hyperandrogenism.

Parameter	ESR1 rs3020314	Mean ± SEM	*P*	ESR1 rs2077647	Mean ± SEM	*P*	ESR1 rs1884051	Mean ± SEM	*P*
Glucose 0′ (mg/dl)	CC	90.21 ± 2.96	0.174	CC	89.79 ± 2.36	0.142	AA	94.63 ± 3.30	0.164
	CT	86.98 ± 1.50		CT	95.39 ± 3.80		AG	86.84 ± 1.53	
	TT	94.71 ± 3.49		TT	86.50 ± 2.26		GG	89.25 ± 3.31	
Glucose 60′ (mg/dl)	CC	131.23 ± 6.83	0.837	CC	136.75 ± 7.21	0.695	AA	137.10 ± 5.31	0.606
	CT	131.24 ± 6.45		CT	134.94 ± 5.82		AG	129.08 ± 6.44	
	TT	135.83 ± 5.56		TT	128.68 ± 6.97		GG	131.48 ± 8.36	
Glucose 120′ (mg/dl)	CC	117.87 ± 8.93	0.363	CC	107.33 ± 5.30	0.846	AA	111.10 ± 4.91	0.221
	CT	103.67 ± 4.10		CT	110.27 ± 5.80		AG	101.95 ± 4.01	
	TT	109.90 ± 5.24		TT	106.10 ± 4.47		GG	118.84 ± 10.87	
Insulin 0′ (μIU/ml	CC	19.81 ± 4.11	0.721	CC	26.09 ± 5.64	0.418	AA	27.21 ± 5.99	0.754
	CT	22.56 ± 3.55		CT	28.73 ± 6.97		AG	21.90 ± 3.72	
	TT	27.46 ± 6.35		TT	17.87 ± 3.33		GG	21.90 ± 4.19	
Insulin 60′ (μIU/ml)	CC	115.57 ± 12.73	0.847	CC	147.39 ± 21.09	0.163	AA	133.85 ± 15.35	0.948
	CT	132.97 ± 13.66		CT	143.43 ± 14.30		AG	130.50 ± 14.36	
	TT	133.76 ± 16.59		TT	106.71 ± 15.43		GG	122.72 ± 12.90	
Insulin 120′ (μIU/ml)	CC	123.08 ± 17.18	0.139	CC	113.23 ± 20.52	0.069	AA	89.28 ± 13.70	0.163
	CT	70.87 ± 10.15		CT	91.71 ± 12.45		AG	73.54 ± 10.49	
	TT	92.69 ± 14.63		TT	62.94 ± 11.46		GG	130.24 ± 19.12	
AG	CC	1.14 ± 0.03	0.173	CC	1.06 ± 0.06	0.256	AA	1.12 ± 0.04	**0.049**
	CT	0.97 ± 0.07		CT	1.12 ± 0.05		AG	0.95 ± 0.06	
	TT	1.10 ± 0.04		TT	0.99 ± 0.06		GG	1.16 ± 0.03	
VF	CC	725.50 ± 155.78	0.298	CC	878.88 ± 279.73	0.061	AA	1076.70 ± 164.06	0.225
	CT	686.07 ± 170.90		CT	1165.09 ± 173.25		AG	655.36 ± 165.11	
	TT	1068.23 ± 170.27		TT	544.14 ± 155.11		GG	678.33 ± 209.97	
BMI	CC	28.77 ± 2.74	0.948	CC	29.51 ± 2.75	0.251	AA	29.36 ± 1.61	0.993
	CT	29.85 ± 1.96		CT	30.99 ± 1.64		AG	29.47 ± 1.94	
	TT	29.16 ± 1.65		TT	26.51 ± 1.85		GG	28.96 ± 3.35	

*AG* distribution of android and gynoid fat, *VF* visceral fat indication, *BMI* Body Mass Index.

Significant values are given in bold.

**Table 6:** Analysis of bone metabolism parameters in relation to ESR1 gene polymorphisms in patients with hyperandrogenism.

Paramater	ESR1 rs30220314	Mean ± SEM	P	ESR1 rs2077647	Mean ± SEM	P	ESR1 rs1884051	Mean ± SEM	P
BMD total	CC	1.19 ± 0.03	0.870	CC	1.18 ± 0.04	0.285	AA	1.20 ± 0.02	0.770
	CT	1.18 ± 0.03		CT	1.21 ± 0.02		AG	1.18 ± 0.03	
	TT	1.20 ± 0.02		TT	1.16 ± 0.02		GG	1.23 ± 0.06	
BMD L1-L4	CC	1.23 ± 0.05	0.923	CC	1.20 ± 0.05	0.255	AA	1.22 ± 0.03	0.951
	CT	1.23 ± 0.02		CT	1.25 ± 0.02		AG	1.23 ± 0.02	
	TT	1.22 ± 0.03		TT	1.20 ± 0.03		GG	1.23 ± 0.06	
T-score	CC	0.70 ± 0.47	0.868	CC	0.24 ± 0.53	0.168	AA	0.41 ± 0.22	0.907
	CT	0.52 ± 0.27		CT	0.80 ± 0.19		AG	0.53 ± 0.27	
	TT	0.40 ± 0.23		TT	0.15 ± 0.28		GG	0.67 ± 0.66	
Z-score	CC	0.37 ± 0.41	0.748	CC	0.25 ± 0.38	0.987	AA	0.10 ± 0.22	0.690
	CT	0.34 ± 0.24		CT	0.23 ± 0.23		AG	0.38 ± 0.24	
	TT	0.11 ± 0.22		TT	0.18 ± 0.25		GG	0.34 ± 0.50	
BMC (g)	CC	2366.33 ± 95.25	0.829	CC	2391.58 ± 90.62	0.176	AA	2430.10 ± 35.47	0.666
	CT	2390.45 ± 64.48		CT	2458.00 ± 33.49		AG	2371.45 ± 62.66	
	TT	2421.24 ± 35.55		TT	2322.38 ± 59.19		GG	2378.20 ± 115.75	
TBS	CC	1.46 ± 0.07	0.529	CC	1.31 ± 0.06	0.279	AA	1.36 ± 0.03	0.529
	CT	1.37 ± 0.03		CT	1.39 ± 0.02		AG	1.37 ± 0.03	
	TT	1.36 ± 0.03		TT	1.36 ± 0.04		GG	1.46 ± 0.07	

*BMC* bone mineral content, *TBS* trabecular bone score, *BMD L1-L4* bone mineral density L1-L4, *T-score* the ratio of the bone mineral density (BMD) of the test person to the average bone density of the young person, *Z-score* bone mineral density index, *BMD total* bone mineral density total.

The presence of the CC genotype for the rs3020314 polymorphism occurring with the lowest frequency was associated with a significantly higher concentration of free estradiol and 17-OH-progesterone compared to the other genotypes. For the CC genotype, a statistical trend was also observed concerning the higher concentration of estradiol (p = 0.053) and androstenedione (p = 0.087) compared to the other genotypes (Tables 2 and 3). Levels of glucose and insulin in women with hyperandrogenism in relation to the distribution of genotypes of the rs3020314 polymorphism did not show any significant statistical differences. The concentration of other parameters: prolactin, LH, FSH, testosterone, vitamin D, vitamin 25(OH)D, calcitonin, parathyroid hormone, osteoprotegerin, sRANKL, leptin and other parameters: BAT%, FAI%, BMD L1-L4, T-score, Z-score, BMD total, BMI, BMC, AG, VF and TBS in relation to the distribution of genotypes of the ESR1 rs3020314 polymorphism did not show any significant statistical differences.

The most common CT genotype for the rs2077647 polymorphism was associated with a significantly higher concentration of DHEA-SO4 compared to the other genotypes. There was also a statistical trend for the highest level of VF in the CT genotype compared to the CC and TT genotypes. In contrast, the CC genotype was associated with the significantly lowest level of vitamin 25-(OH)D compared to the CT and TT genotypes. There was also a statistical trend related to the CC genotype for the highest concentration of free estradiol (p = 0.095) and the highest concentration of insulin at 120 min. (p = 0.069) compared to the CT and TT genotypes (Tables 3 and 5).

The presence of the mutant GG genotype for the rs1884051 polymorphism occurring with the lowest frequency was associated with a significantly higher concentration of free estradiol and 17-OH-progesterone compared to the AA and AG genotypes. The android to gynoid fat decomposition ratio was also significantly higher for the GG genotype. For the GG genotype, there is also a statistical trend regarding a higher concentration of androstenedione (p = 0.096) compared to the AA and AG genotypes. For the AA genotype, the existence of a statistical trend was also shown, related to a higher concentration of estradiol (p = 0.055) compared to the AG and GG genotypes (Tables 2 and 3)**.**

## Discussion

Estrogens affect the bone mineralization process through the ER-α receptor, which is encoded by the ESR1 (Estrogen Receptor-1) gene^31^. Activation of the receptor depends on the intracellular level of the free estrogen fraction, receptor affinity, and the number and sensitivity of estrogen receptors. The level of bioavailable estrogen, which varies with age, has been shown to be an independent predictor of bone mineral density in both men and women. Estrogen deficiency is associated with a decrease in bone mineral density and bone loss in both sexes, also related to age^32^. The ESR1 gene encoding the ER-α receptor may have many polymorphic variants. Some polymorphisms, such as XbaI (rs9340799) and PvuII (rs2234693), have long been identified as affecting bone mineral density and are associated with an increased risk of osteoporosis. However, research results are often controversial and inconclusive. The frequency of specific polymorphisms is additionally differentiated also ethnically^33^. In their analysis, Wang et al. emphasize the role of ESR1 polymorphisms in bone and articular cartilage diseases and that estrogen concentration may be a predictor of bone mineral density and the risk of osteoporotic fractures^34^. A meta-analysis by Ioannidis et al. showed that the XbaI (rs9340799) polymorphism is significantly related to the mineral density of the femoral neck and the lumbar spine. However, the importance of the PvuII polymorphism (rs2234693) for BMD in the lumbar spine and the femoral neck has not been confirmed^35^. On the other hand, studies by Albagh et al. showed no significant correlation between the polymorphisms of the ESR1 XbaI (rs9340799) and PvuII (rs2234693) genes and bone mineral density. When polymorphisms were analyzed individually, a significant relationship was shown between specific haplotypes and BMD^24^.

The conducted studies were aimed at determining the influence of three gene polymorphisms: ESR1 rs1884051, rs2077647 and rs30220314 on selected metabolic, hormonal and bone metabolism parameters in 80 young women with hyperandrogenization. Studies by many authors have shown a relationship between the occurrence of specific ER-α receptor polymorphisms with bone remodeling, fracture resistance or the risk of osteoporosis. Bander et al. demonstrated a significant relationship between the occurrence of ESR1 rs1884051 and rs2077647 polymorphisms with osteoporosis in patients with HIV infection, with men dominating in the study group^36^. However, Jin Shu et al. did not show a relationship between the ESR1 rs2077647 polymorphism and postmenopausal osteoporosis in the Chinese population^37^. Studies by Kurt et al. also did not show a relationship between the occurrence of osteoporosis and the polymorphisms of the ESR1 rs9340799 and rs2234693 gene. However, a correlation between the occurrence of a lower BMD in the necks of the femurs in the population of Turkish postmenopausal women has been demonstrated^38^. He et al. showed that the rs2234693 polymorphism is also significantly associated with premature ovarian failure (POF)^39^.

Numerous studies have shown a positive correlation of ESR1 and ESR2 gene polymorphisms with polycystic ovary syndrome (PCOS) in Caucasian women and in the Chinese, Iranian, Pakistani, Greek and Brazilian populations^40^. The polymorphisms of these receptors appear to be good markers for PCOS and metabolic disorders. PCOS is a complex disorder with multifactorial pathogenesis affecting women of reproductive age, characterized by the occurrence of, inter alia, non-ovulatory cycles, hyperandrogenism, obesity, insulin resistance, increased risk of type 2 diabetes and other metabolic disorders. A study by Douma et al. conducted on a population of 254 Tunisian women with PCOS showed a relationship between ESR1 (s2234693, rs9340799, rs3798577, rs3020314) and ESR2 (rs1256049) gene polymorphisms with PCOS. A relationship has been found with the LH concentration, LH /FSH ratio or hyperandrogenism, and especially with the parameters of the metabolic syndrome (rs9340799) and hyperglycemia (rs3798577)^40^. In a study conducted in a group of 170 young women aged 12–25 years diagnosed with PCOS, Pereira-Eshraghi et al. did not show a relationship between the level of free and total testosterone, or the presence of PCOS on bone mineral density. On the other hand, obesity and insulin resistance turned out to be an independent predictor of decreased BMD in the cervical and femoral neck^41^. Many previous studies have shown the effect of PCOS on BMD^42,43^. Another study conducted by Adami et al. showed a positive correlation between BMD in the femoral neck and spine with the concentration of androstenedione and free testosterone in patients with PCOS^43^. Glintborg et al. demonstrated that the level of free testosterone and insulin was significantly higher in female hirsutism patients of reproductive age than in the control group and was positively correlated with BMD in the cervical and spine. A significantly lower level of vitamin 25-(OH)D3 was also demonstrated in patients with hirsutism compared to the control group. However, in these studies, the study group consisted of only 51 women^44^. Katulski et al. demonstrated that the adverse effect of estrogen deficiency on mineral density in PCOS patients is not outweighed by higher androgen production and they showed lower BMD compared to the control group. It was also shown that BMD in the lumbar spine was significantly lower in the PCOS group than in the control group. The study group included 69 women with PCOS, aged 17–34^45^. Karadağ et al. observed in a study of 103 PCOS patients that despite the positive effects of hyperandrogenemia and hyperinsulinemia on BMD, patients had lower bone mineral density in the lumbar spine and femoral neck compared to the control group, which was due to decreased estrogen levels. BMD was positively correlated with the concentration of total testosterone and androstenedione, and higher in PCOS patients with hyperanrogenemia compared to patients with normal androgen levels^46^. The discrepancies observed in the test results can probably be explained by the different size of the groups studied, different inclusion criteria, differences in the ages of the patients, or the intensity and type of symptoms. Kuźbicka et al. demonstrated the relationship of selected polymorphisms of the ESR1 gene (rs2234693, rs6902771, rs7774230) with the occurrence of metabolic syndrome in postmenopausal women^47^. The ESR1 gene polymorphisms (rs2234693, rs9340799, rs2077647) also play a role in the exposure of the fetus to estrogens^48^.

This research is a step towards explaining the influence of different ERα receptor variants on bone metabolism, metabolic and hormonal parameters in young women with hyperandrogenization.

However, a relatively small number of respondents, the lack of control group are potential limitations of the study.

A larger cohort study will be necessary extended to include studies of additional markers of bone turnover to determine the risk of mineralization disorders in young women with hyperandrogenism.

Our studies showed the relationship between the concentration of free estradiol (FEI) and the concentration of 17-OH-progesterone, and the rs3020314 and rs1884051 polymorphisms of the ESR1 gene. The correlation between the concentration of DHEA-SO4 and vitamin 25-(OH)D with the rs2077647 polymorphism was also demonstrated. On the other hand, the distribution of android and gynoid fat—the AG coefficient was associated with the rs1884051 polymorphism of the ESR1 gene.

In conclusion, the ESR gene polymorphisms may be associated with hormonal disturbances in the concentration of estrogens and androgens, in hyperandrogenism in young women which may indirectly affect bone mineral density. However, no statistically significant relationships were found between the studied polymorphisms and the selected parameters of mineral metabolism.
